# Development of Nanomaterials to Target Articular Cartilage for Osteoarthritis Therapy

**DOI:** 10.3389/fmolb.2022.900344

**Published:** 2022-08-11

**Authors:** Chenyu Rao, Sirong Shi

**Affiliations:** State Key Laboratory of Oral Diseases, West China Hospital of Stomatology, Sichuan University, Chengdu, China

**Keywords:** osteoarthritis, drug delivery system, targeted nanomaterials, articular cartilage, synovium

## Abstract

Osteoarthritis (OA) is an obstinate, degradative, and complicated disease that has drawn much attention worldwide. Characterized by its stubborn symptoms and various sequela, OA causes much financial burden on both patients and the health system. What’s more, conventional systematic therapy is not effective enough and causes multiple side effects. There’s much evidence that nanoparticles have unique properties such as high penetration, biostability, and large specific surface area. Thus, it is urgent to exploit novel medications for OA. Nanomaterials have been sufficiently studied, exploiting diverse nano-drug delivery systems (DDSs) and targeted nano therapeutical molecules. The nanomaterials are primarily intra-articular injected under the advantages of high topical concentration and low dosage. After administration, the DDS and targeted nano therapeutical molecules can specifically react with the components, including cartilage and synovium of a joint in OA, furthermore attenuate the chondrocyte apoptosis, matrix degradation, and macrophage recruitment. Thus, arthritis would be alleviated. The DDSs could load with conventional anti-inflammatory drugs, antibodies, RNA, and so on, targeting chondrocytes, synovium, or extracellular matrix (ECM) and releasing the molecules sequentially. The targeted nano therapeutical molecules could directly get to the targeted tissue, alleviating the inflammation and promoting tissue healing. This review will comprehensively collect and evaluate the targeted nanomaterials to articular cartilage in OA.

## Introduction

Epidemiologically, the outbreak of osteoarthritis (OA) shows a prejudice of females in gender and the age more than 65 (Felson et al., 1987; Xia et al., 2014; Szilagyi et al., 2022). Clinical symptoms vary among patients. Knee damage displays principally, followed by hip and hand joints (Ummarino et al., 2020). Evidence reveals that obesity, trauma, and genetic factors are related to the appearance and development of OA (Vina and Kwoh, 2018; Liu et al., 2021). Obesity could not only metabolically impact OA with a higher level of inflammatory factors but also increase the burden of the knee and hip joint, resulting in chronic mechanical damage, which means further progress of OA (Vina and Kwoh, 2018; Ummarino et al., 2020). Joint trauma caused by intense sports, accidents, and surgeons could also assist the OA pathology (Antoni et al., 2021). Congenital deficiency, including joint dysplasia and deformity, shows a susceptible trend towards the disease, attributed to abnormal joint stress and secondary mechanical damage (Vina and Kwoh, 2018).

Conventionally, the administration of OA is classified into two categories, including medication and surgeon (Abramoff and Caldera, 2020; Ummarino et al., 2020). The current clinical drug treatment mainly refers to non-steroidal anti-inflammatory drugs and corticosteroids (Abramoff and Caldera, 2020). However, non-steroidal anti-inflammatory drugs have obvious adverse reactions involving the gastrointestinal tract, cardiovascular and cerebrovascular (Pelletier et al., 2016). While long-term use of corticosteroids may cause osteoporosis, hypertension, diabetes, etc., (Wernecke et al., 2015). Furthermore when the above two drugs are taken orally, due to the clearance of the drugs by the liver and plasma, higher doses are required to achieve effective blood drug concentrations, which further increases the possibility of adverse reactions (Wernecke et al., 2015; Pelletier et al., 2016; Abramoff and Caldera, 2020; Ummarino et al., 2020).

To overcome the defects above, we need to figure out a novel drug administration approach based on higher local concentration and lower systemic dosage. Thus, the efficacy would be enhanced while the adverse effects would be alleviated. Nanotechnology has been widely exploited, introducing various nanomaterials with unique properties such as high permeate, specifical and long endurance *in vivo* or *in vitro* (Brown et al., 2019; Zhang T. et al., 2020; Ma et al., 2022). Plenty of evidence shows that nanomaterials could be therapeutic molecules combining with targeted tissue directly and be drug delivery systems (DDSs) transporting the drug molecules specifically (Brown et al., 2019; Ummarino et al., 2020). In addition, previous studies have demonstrated that nanomaterials have multiple bio-functions (Zhang et al., 2021) [anti-tumor (Ma et al., 2022), anti-inflammation (Wang et al., 2022; Zhang et al., 2022), differentiation promotion (Li S et al., 2021), immune modulation (Qin et al., 2022), and neuro-protection (Zhou et al., 2021; Zhu et al., 2022)]

This manuscript comprehensively summarizes the current OA-targeted nanodrugs, classified by their targeted tissues, including chondrocytes, the cartilage extracellular matrix (ECM), synovium matrix, and synovial cells. We also distinguish the nanodrugs into nano-therapeutic molecules that directly react with their target and DDSs that specifically transport the drug molecules embedded in them.

## Structure of Articular Cartilage and Synovium

### Chondrocytes

As is known to all, the joint undertakes the burden of sports with a series of physiological bases such as articular cartilage, synovium, and ligaments. Articular cartilage consists of chondrocyte and ECM, being the major portion of degradation caused by OA, meanwhile the important target of the therapeutic molecules.

Generally, chondrocytes participate in osteogenesis via the process of proliferation and terminal hypertrophic alternation. Previous investigation has proven that chondrocytes are derived from mesenchymal stem cells (MSCs) at the embryonic stage, followed by hypertrophic change (Kronenberg, 2003). The hypertrophic chondrocytes are endowed with various functions, including osteogenesis and bone mineralization. The spatial distribution of chondrocytes is characterized by its regular occurrence in cartilage lacuna and elimination at the chondro-osseous junction. Several scholars have studied that the post-mature chondrocytes are likely to differentiate into osteoblasts and keep the promising potency by differentiating into multiple lineages (Yang et al., 2014).

**FIGURE 1:**
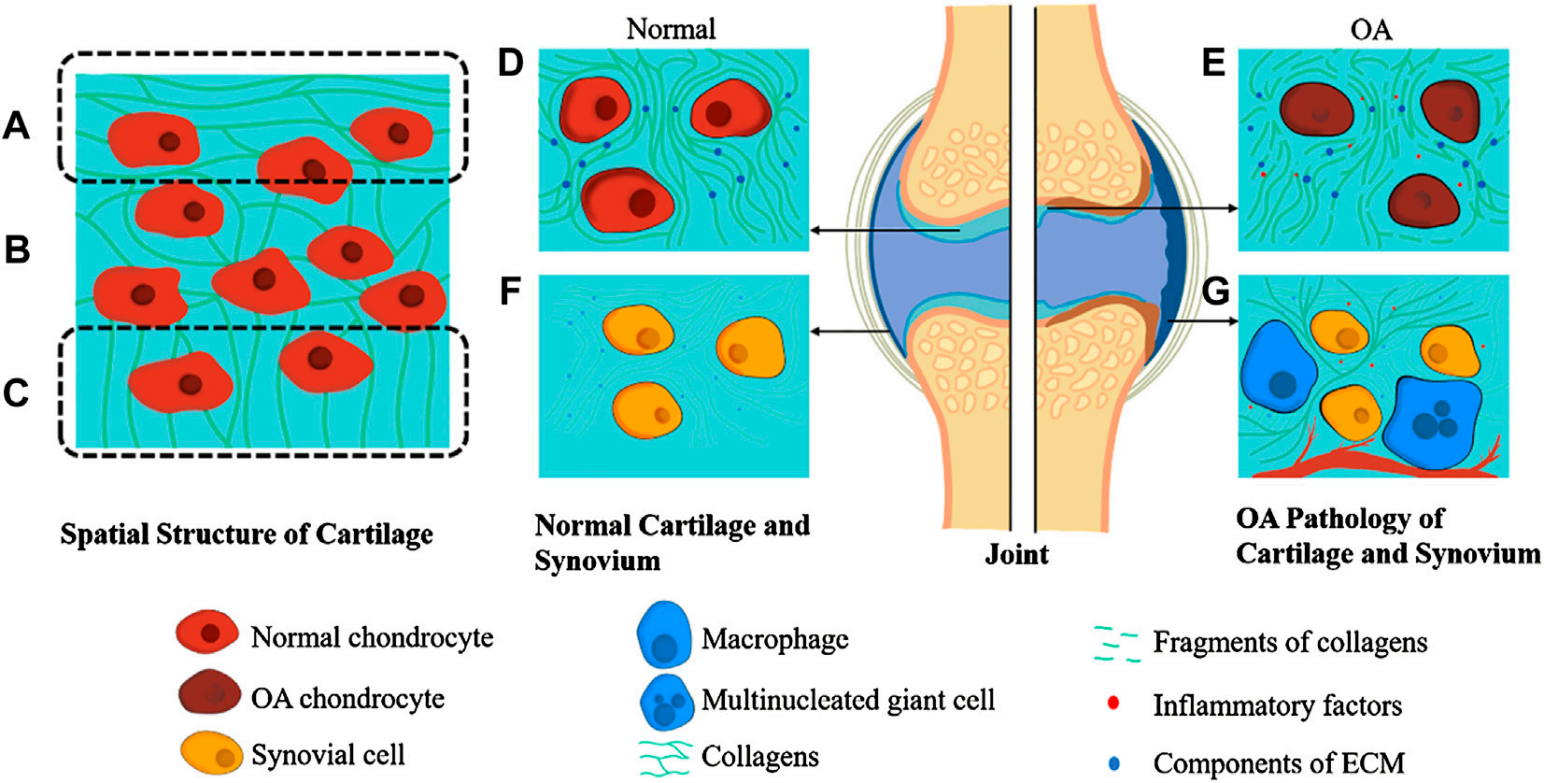
**Pathology of OA Joint and Spatial Structure of Cartilage.** The spatial structure of cartilage is divided into three parts. The collagens in superficial zone are aligned parallel to the tissue surface **(A)**. At the same time, they are randomly orientated in the middle zone **(B)** and are perpendicular to the interface of cartilage and subchondral bone in deep zone **(C)**. Normal articular cartilage contains chondrocytes and ECM full of collagens, proteoglycans, and glycosaminoglycans **(D)**. In contrast, the OA cartilage is recognized by the apoptosis of chondrocytes and ECM fragments **(E)**. Normal synovium consists of synovial cells and synovial ECM, which is similar to cartilage **(F)**. At the same time, the OA synovium is characterized by infiltration of macrophages, multinucleated giant cells, fibrosis, and angiogenesis **(G)**.

The exclusive presence of chondrocytes in the ECM makes articular cartilage devoid of vascular vessels and nerves. The fluid nourishes chondrocytes from synovium and subchondral bone (Figure 1D) (Armiento et al., 2018; Messina et al., 2019). With the stagnation of the premature stage, chondrocytes intervene in the perpetual balance of articular cartilage vitally (Komori, 2020). Chondrocytes synthesize and secrete collagen, polysaccharides, and their derivatives to construct ECM.

### ECM

ECM is the essential part of articular cartilage to accommodate chondrocytes, composed of fibers, proteoglycans, and glycoproteins (Carballo et al., 2017). The spatial distribution of cartilage ECM varies overtly, which means five zones of ECM with diverse components arranged respectively from surface to depth (Brown et al., 2019).

Collagen is the domaining fiber constituting the ECM, classified as collagen II (the predominant one), III, IV, VI, IX, X, and XI (COL-II, III, IV, VI, IX, X, XI), endowing the mechanical properties of cartilage such as tenacity and flexibility (Wilusz et al., 2014; Carballo et al., 2017). Previous studies show that COL-II takes up about 90% volume of fibrils and interacts with COL-IX and XI, and further becomes coarser and cross-linked with the help of leucine-rich proteoglycans and cartilage oligomeric matrix protein (Brown et al., 2019). COL-IV constructs micro-fibrils distributed around chondrocytes, endowing the ECM with the elastic mechanical property. The COL-IV and X don’t form macromolecular fibrous structures, mainly located at tangential and calcified zones (van der Kraan et al., 2001; Foldager et al., 2014). Chondrocytes merely take up about 2% volume of the articular cartilage, involving the formation of ECM with the regular releasement of inflammatory and growth factors while maintaining the cartilage homeostasis to keep it healthy (Carballo et al., 2017).

Proteoglycans and glycoproteins are also the main components of cartilage ECM. Glycoproteins constitute proteoglycans via combining glycosaminoglycans (GAGs) on their sidechains no less than three. GAGs are bioactive and comprise chondroitin sulfate (ChS), dermatan sulfate (DS), heparan sulfate (HS), keratan sulfate (KS), and hyaluronic acid (HA) (Brown et al., 2019). Aggrecans have the largest quantity in proteoglycans of ECM, followed by perlecan and laminin (Maroudas, 1976; Brown et al., 2019). Aggrecans can form aggregates as core protein via conjugating with HA molecules, thereby exerting their bio-function in the reticulated structure of collagens (Schneevoigt et al., 2017; Han et al., 2019). What’s more, aggrecans can also endow cartilage with the property of elasticity through the associated water molecules (Schulz and Bader, 2007).

### Synovium

The physiological functions of synovium comprising joint cavity formation, fluid dynamic accommodation, and chondrocytes’ nutritional transport in an articulation mustn’t be ignored. Synovium is also a vital component of a joint (Figure 1F) (Mathiessen and Conaghan, 2017). There are two layers constituting normal synovium. The outer one is called subintima, characterized by the vascular and lymphatic vessels within it, while the inner one is identified by its macrophages and fibroblasts (Smith, 2011; Mathiessen and Conaghan, 2017). Taking burdens of immune reactions, macrophages widely exist in the synovium. In other words, macrophages phagocytose and polarize, thus proceed an inflammatory process. As a cyst-like structure formed with synovium, the synovial cavity contains a small amount of synovial fluid abundant with HA and water.

### Spatial Structure of Cartilage

The spatial structure of articular cartilage is complicated and orderly, divided into three parts from superficial to deep (Figures 1A–C): the superficial zone where the collagen fibrils are aligned parallel to the tissue surface, the middle zone where the collagen fibrils orientation is random, and the deep zone that is adjacent to subchondral bone in which the fibrils are perpendicular to the interface (Carballo et al., 2017). Previous studies have shown that the number of molecules contained in articular cartilage varies from region to region. The middle zone has the largest charge because it contains the most negatively charged proteoglycan. In contrast, the collagen fibers have the highest content in the superficial zone, giving it a higher function of shear resistance (Mow and Guo, 2002; Carballo et al., 2017).

## Pathology of Osteoarthritis

OA is an obstinate disease characterized by degradation and various disorders such as joint pain, impaired mobility, and inflammation, with much attention to public health authorities and governments worldwide (Reddi et al., 2011; Bottini et al., 2016; Shah et al., 2020). It is reported that 151 million individuals have been affected by the disease, leading to a fiscal burden of $185.5 billion annually in the United States alone (Brown et al., 2019). OA has become a socioeconomic problem impacting the residents worldwide due to its increasing morbidity (Hootman and Helmick, 2006). It has been widely explored that the risk factors of OA mostly ascribe to family inheritance, senescence, overweight and joint trauma, whereas the pathogenesis of OA is still largely vague (Xia et al., 2014). Currently, it is widely known that OA is a complicated process in which severe degradation of articular cartilage is the most notable. The whole joint is influenced during OA, impairing the synovium, joint ligaments, and subchondral bone (Abramoff and Caldera, 2020).

### ECM Degradation

Degradation of the ECM might appear first during the progression of OA (Goldring and Goldring, 2010; Mobasheri and Batt, 2016) (Figure 1E). First, the various metalloproteinases and aggrecanases in ECM will increase abnormally, and increase the catabolism of ECM, and cause the gradual degradation of macromolecular substances such as collagen fibers that maintain the stable structure of ECM, triggering the destruction of structure and function of ECM (Bottini et al., 2016).

Furthermore, ECM is damaged by the inflammatory cytokines (IL-1, IL-6, and TNF), reactive oxygen species (ROS), and peroxynitrite released by various cells at the lesion (Bottini et al., 2016). Matrix metalloproteinases (MMPs) are a family (Winer et al., 2018), relying on Ca^2+^, Zn^+^ which are the cofactors to exert their function of matrix resolution (Wang and Khalil, 2018; Kou et al., 2021). Scholars have classified MMPs into 26 categories, numbered MMP1∼26. The substrates of MMP are almost all the protein components of ECM, so scientists divide it into collagenase, gelatinase, matrix-degrading, and so on according to the different substrates (Cui et al., 2017). ROS is a one-electron reduction product of oxygen, including superoxide anion, hydrogen peroxide (H_2_O_2_), and hydroxyl radicals (Turrens, 2003; Brieger et al., 2012). ROS are detrimental to the components of ECM via amino acid modification, peptide breakage, protein oxidative allostery, and undermining the structure of macromolecules (Jakubczyk et al., 2020).

### Chondrocytes Alteration

The enhanced catabolism of ECM leads to an increase in the content of matrix fragments and chemokines, which in turn causes a series of changes in the phenotype of chondrocytes (Bottini et al., 2016). Chondrocytes have various manifestations of hypertrophy, terminal differentiation, and apoptosis (Mobasheri and Batt, 2016). Generally, they show a tendency to repair the damage.

In the second stage of OA, chondrocytes are stimulated by arthritis (Figure 1E). Chondrocytes upregulate the secretion of catabolic enzymes and mediums, enhancing cartilage degradation, and further promoting the apoptosis of chondrocytes themselves. Some evidence shows that the alternation of ECM in OA precedes the changes of osmolality and ionic microenvironment, leading to dynamic adjust of chondrocytes (Jeremiasse et al., 2020).

### Synovium Inflammation

OA can cause synovium inflammation (Figure 1G). Some scholars reckon that synovial membranes and fluid are the keys between systematic inflammation and OA. Topical arthritis is mainly brought by synovium and synovial fluid via synthesizing and releasing inflammatory factors (IL-1, IL-6, TNF) and MMPs. Leading to catabolic loop and cartilage damage, synovial fluid can also be a biochemical pathway to unravel the complicated molecular mechanism of OA (Bottini et al., 2016; Ingale et al., 2021). Many of the synovial cells are macrophages, the innate immune cells derived from mononuclear leucocytes (Falconer et al., 2018). Macrophages exert the inflammatory response via the polarization toward pro-inflammatory (M1) and anti-inflammatory (M2) phenotypes (Muñoz et al., 2020). In the context of early-stage arthritis, the macrophages in the synovium are stimulated by inflammatory factors such as LPS, IFN-γ, and TNF-α, thus polarizing towards the M1 phenotype (Cook et al., 2003). The M1 cells would release even more inflammatory factors, including IL-6, IL-12, IL-23, and TNF-α. Not only that, ROS, NO, MMP-1,3,13 secreted by M1 cells can harm articular cartilage directly (Nagai et al., 2003; Haltmayer et al., 2019; Jakubczyk et al., 2020). The inflammatory and damage factors mentioned above will recruit more leucocytes, including mononuclear cells and macrophages, sequentially promote their proliferation and differentiation towards M1 phenotype macrophages, creating a vicious cycle (Culemann et al., 2019).

## Therapeutic Administration

### Route of Administration

Drug molecules need to reach the lesion to perform the corresponding pharmacological functions, thereby alleviating symptoms and curing diseases. There are many ways to administer medicine in the medical profession. The common ones are oral, subcutaneous injection, intravenous injection, sublingual administration, etc. As for the drug treatment of OA, the routes can be divided into two major groups, including systematic treatment and topical treatment.

Systematic treatment of OA consists of oral and injection administration with conventional anti-inflammatory medicine such as NSAIDs, capsaicin, weak opioids, and narcotic analgesics. It is more conventional but less effective and targeting (Abramoff and Caldera, 2020).

The topical treatment of OA is intra-articular injection. Compared with systemic administration, intra-articular injection administration has the advantages of small systemic side effects, high local drug concentration, and long drug retention time. Local injection of hormones and anti-inflammatory drugs can stabilize cell membranes, reduce inflammatory exudation and relieve symptoms (Altman and Barthel, 2011; Brown et al., 2019). Notwithstanding the universal application of intra-articular injection with the drugs mentioned above, the blemishes such as rapid drug diminishment and apparent off-target effects of the drugs are still unignorable (Brown et al., 2019). In recent years, intra-articular injection of nano-medicine has gradually entered the public eye. Many scholars have tried to use nano-medicine. Many nano-DDSs and nano-medicine have been exploited because of their strong penetration, high retention, and targeting properties.

### Conventional Medicine Has Obvious Defects

Destruction of the articulation is the most obvious feature during the OA process, resulting in multiple clinical symptoms, including inflammation, pain, and restricted movement of joints (Abramoff and Caldera, 2020). To relieve symptoms and eliminate the disease, scholars have recommended various treatment methods of which nonpharmacologic, pharmacologic, and surgical therapy (Taruc-Uy and Lynch, 2013). Paracetamol, other non-steroidal anti-inflammatory drugs (NSAIDs), and capsaicin are topically utilized to treat OA, leading to a wide range of adverse events (AEs) such as hepatotoxicity, renal damage, and cardiovascular injury. Meanwhile, a relatively minimal therapeutic effect (Altman and Barthel, 2011; Taruc-Uy and Lynch, 2013). Paracetamol is a universally used analgesic-antipyretic, leading to hepatotoxicity at a dose of 3.25 g/day, which merely causes little therapeutic effects. NSAIDs have extensive AEs on digestive, cardiovascular, and urinary systems, relying on the dose, duration, and age factors (Altman and Barthel, 2011). Capsaicin can also have AEs such as irritation, nerve degeneration, and occasional coughing (Mason et al., 2004; Gibbons et al., 2010; Altman and Barthel, 2011). Thus, conventional pharmacological treatment emerges with its defective aspect of high risks of heterogeneous side effects and low safe dose. Therefore, we need to find new drugs that can target therapy to increase the local drug concentration while reducing the dose, achieving the goal of increasing efficacy and reducing systemic reactions. Therapeutic nanoparticles (also called nanodrugs) have gradually been recognized by the medical field, with unsurpassed targeting, membrane penetrability, thus could lower the risk brought by high plasma concentration (Brown et al., 2019).

### Nanoparticles Have Diverse Unique Properties

The size of particles significantly affects their physical, chemical, and biological properties. The size of drug molecules can even directly affect its pharmacological properties, enhancing its therapeutic effects, such as anti-tumor, anti-angiogenic, anti-inflammatory, and proliferation promoting functions. Scholars endow the medicine with a lower dosage and adverse effects rate at the nanoscale. Nanodrugs and nano-DDSs possess specific traits likely to be attributed to their properties of nanoparticles elaborated below.

The particles’ size and specific surface area affect their biological properties, including the particles’ penetration, distribution, and clearance after entering the body. The smaller the particle size and the larger the specific surface area, the more likely it will cause adverse reactions. Nanoparticles may damage normal tissue by generating free radicals, an effect that intensifies as the size of the nanoparticles decreases (Gatoo et al., 2014). Some studies have also suggested that nanoparticles smaller than 50 nm have strong penetration and can be distributed in almost all tissues, resulting in enhanced side effects (De Jong et al., 2008; Gatoo et al., 2014).

*In vitro* experiments have shown that nanoparticles with a size of about 100 nm are most easily endocytosed by cells (Xu et al., 2012; Liu et al., 2017). This might be attributed to a better combination and absorption of surface cluster receptors resulting from nanoparticles with a size of 100 nm. Thus the particles trigger invagination of the cell membrane to form vesicles and mediate endocytosis (Liu et al., 2017). In addition, studies have shown that nanoparticles *in vivo* tend to adsorb various molecules on the surface to form protein coronas (PCs), thus the size of the particles interacting with the cell membrane is often larger than the size of the nanoparticles just composited (Walkey et al., 2012; Sun et al., 2013). There is evidence that the optimal nanoparticle size for mediating endocytosis *in vivo* is less than 34 nm (Choi et al., 2010).

Nanoparticles have different morphologies after synthesis, including spherical, rod-shaped, tubular, and tetrahedral (Gatoo et al., 2014). Different shapes of nanoparticles have different effects on triggering endocytosis (Liu et al., 2017). Previous studies have confirmed that spherical nanoparticles better mediate endocytosis and are less toxic (Champion and Mitragotri, 2006; Lee et al., 2007). At the same time, the higher the aspect ratio and the more elongated morphology of nanoparticles, the greater their potential toxicity, which may be because the longer fibers are not easily cleared by macrophages, causing inflammation in the tissue (Fubini et al., 2011; Gatoo et al., 2014).

Free radicals, metal ions, etc. on the surface of nanoparticles can lead to the generation of ROS, triggering different degrees of toxic reactions, while specific modifications on the surface of nanoparticles (such as hydrophilic polyethylene glycol and other surface-active copolymers) can reduce the side effects and enhance the stability of nanoparticles (Liu et al., 2017).

The charge on the surface of nanoparticles can also affect their function. For example, positively charged nanoparticles have higher clearance rates but also have a better affinity for certain negatively charged biological structures (Gatoo et al., 2014; Liu et al., 2017; He et al., 2020).

The hydrophobicity of the surface of nanoparticles can also affect the efficiency of their cellular uptake. Hydrophobic molecules are more likely to enter the cell membrane and be endocytosed by cells; nanoparticles with a rough surface have a stronger interaction with cells, making it easier to enter cells and mediate endocytosis (Gatoo et al., 2014).

## Cartilage Targeted Nanodrugs

### Chondrocytes Targeted Nanodrugs

**FIGURE 2:**
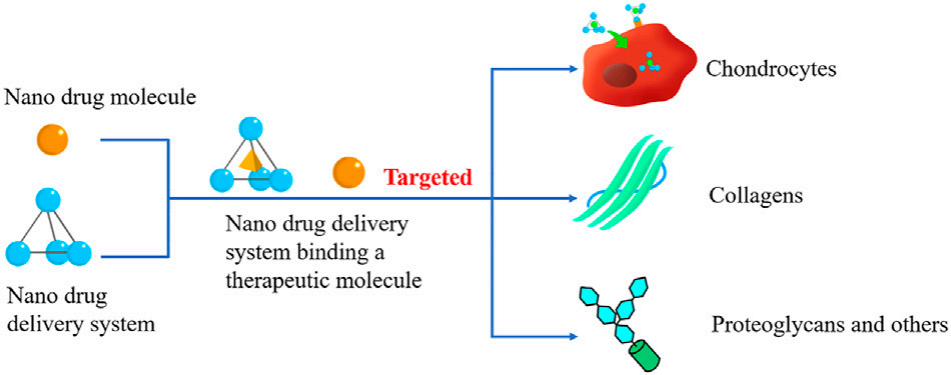
Cartilage Targeted nanodrugs. Cartilage-Targeted nanodrugs can be divided into nano therapeutical molecules and nano-drug delivery systems (DDSs). Both of them can specifically react with the components of cartilage, mediating the OA treatment procedure.

Chondrocytes undertake the burden of the generation of ECM and renewal of its components, attributed to their unique existence in ECM (Figure 1D). Researchers have investigated a variety of nanodrugs to treat OA to ease the symptoms, eliminate the cause of the disease, and improve the quality of daily life (Figure 2).

Various therapeutic molecules could decline the inflammatory reaction and ease the symptoms of OA. Still, they are restricted by their off-target effects and low concentration in joint. Previous studies have proved that nanoparticles could be designed as a medication delivery system (Table 1). Cationic polyethylenimine (PEI) is a vital approach to delivering drugs to the lesion site. Scholars modified a targeting ligand (Chondrocyte-homing peptide, CAP) on the PEI to reduce the toxicity and dose meanwhile assemble the pharmaceutical molecule (Bottini et al., 2016). Lipid-based carriers are also recognized as a conventional nano-delivery tool to load hydrophobic therapeutic molecules, while polymeric nanoparticles can encapsulate multiple medicines targeting chondrocytes (de Silva et al., 1979). For instance, in 2019, Cho, H. et al. successfully loaded protein kinase D inhibitor (PKDi) onto nanosomes, creating PKDi-Nano. PKDi can specifically ease the inflammatory reaction primarily caused by PKD, while the clinical application is restricted by its off-target effects and low cellular internalization. PKDi-Nano can significantly decline the defects above and reduce the inflammation damage via the nuclear factor kappa-light-chain enhancer of the activated B cells (NF-κB) pathway. The lipid-based nanosome endows PKDi with stability, leading to a relatively long-time NF-κB pathway activation. Thus, the restoration of chondrocytes would be enhanced (Cho et al., 2019). Some scholars synthesized cationic liposomes via film dispersion and loaded microRNA-140, demonstrating that CL@miR-140 could successfully lead to chondrocyte remedy. After the nanomaterial gets to the chondrocytes, the DDS could release and transport the miR-140 into the cells. Thus, the mRNA could specifically upregulate the COL2A1 mRNA, modulating COL-II synthesis (He K et al., 2021). In 2022, Velot É et al. synthesized agro-based rapeseed liposomes carried transforming growth factor (TGF)-β1 (Lipo@TGF-β1) (Velot et al., 2022). The liposomes encapsulated the TGF-β1, endowing it with biostability, a long half-life, and good penetration in cartilage. Then the TGF-β1 would bind with chondrocytes receptors to mediate several signaling pathways (ERK/p-38 MAPK/Smad), which retains the chondrocytes’ articular phenotype. Other nanoparticles can also be used to target chondrocytes. In 2019, Ouyang, Z. et al. (Ouyang et al., 2019) successfully synthesized Gd_2_(CO_3_)_3_ core-based nanoparticles, then anchored a cartilage-targeting peptide and loaded hesperetin (Hes) into the nanoparticles, forming a chondrocyte-targeted drug delivery system called Hes-Gd_2_(CO_3_)_3_@PDA-PEG-DWpeptide (HGdPDW). The scholars demonstrated that HGdPDW could specifically inhibit chondrocyte TLR-2 to alleviate the degeneration via TLR-2/NF-κB/Akt signaling pathway. Exosomes have been exploited for OA therapy as well. In 2017, Cosenza, S. et al. successfully synthesized mesenchymal stem cells derived exosomes and confirmed the protective function of OA cartilage (Cosenza et al., 2017). In 2020, Kim, Y. et al. (Kim et al., 2020) demonstrated that exosomes from mesenchymal stem cells (MSC-exosomes) could be used as a targeting drug delivery system for chondrocytes. The MSC-exosomes could specifically protect chondrocytes via multiple methods, such as downregulating the inflammatory factors secretion, declining the expression of prostaglandin E2 (PGE2), or reducing the binding affinity of transcription factor c-jun activating protein-1 (AP-1) and NF-κB. In 2020, Sirong, S. et al. (Sirong et al., 2020) verified that the tetrahedral framework nucleic acids loaded with wogonin (TFNAs@wogonin) could restrain the inflammation with chondrocytes apoptosis attenuation as well as chondrogenic marker expression enhancement.

**TABLE 1:** Chondrocyte targeted nanomaterials.

Name	Type	Mechanism	Citation
CAP-PEI	Drug delivery system	Utilize the nanoparticles’ inherent properties of high penetration, stability and compatibility to deliver therapeutic molecules	Bottini et al. (2016)
PKDi-Nano		Specifically modified DDSs could obtain targeting	Cho et al. (2019)
CL@miR-140			He K et al. (2021)
Lipo@TGF-β1			Velot et al. (2022)
HGdPDW			Ouyang et al. (2019)
MSC-exosome			Kim et al. (2020)
TFNAs@wogonin			Sirong et al. (2020)
IGF-1	Therapeutic molecule	Promote chondrocytes to synthesize ECM components	(Loffredo et al., 2014; Geiger et al., 2018)
ChS		Enhance chondrocytes proliferation and migration	Hsu et al. (2022)
SeCS		Reduce apoptosis of chondrocytes	Wang et al. (2020)
3′-SL		Inhibit apoptosis, enhance synthesis of chondrocytes	Baek et al. (2021)
RXRα modulator K-80003		Target chondrocyte nuclear receptor RXRα	Li S et al. (2021)
CircSERPINE2		Target miR-1271-5p and ERG	Shen et al. (2019)
Circ_0020093		Inhibit miR-23b	Feng et al. (2021)
CircPDE4B		Regulate p-38/MAPK signaling pathway	Shen et al. (2021)
CYTOR		The knock of CYTOR could reverse anti-OA drug effects, indicating CYTOR to be a promising target drug	Wang et al. (2021)

Several scholars have also shown that nanoparticles can be used directly as therapeutic molecules or as carriers and effectors simultaneously (Table 1). The previous investigation revealed that insulin-like growth factor 1 (IGF-1) could upregulate the synthesis of ChS-rich aggregating proteoglycans even at the relatively high concentration of inflammatory factors such as IL-1 and TNF-α, indicating that IGF-1 has the potential to ease arthritis (McQuillan et al., 1986; Tyler, 1989). In 2014, the heparin-binding (HpB) domain of human EGF was assembled on IGF-1, creating HpB-IGF-1 nanomaterial by Loffredo et al. (Loffredo et al., 2014) The HpB-IGF-1 obtained much more stability than single IGF-1. Geiger, BC et al. (Geiger et al., 2018) also modified the IGF-1 with PAMAM dendrimer, endowing the nanoparticles with higher levels of residence time. In 2014 Jain A et al. (Jain et al., 2014) composited diacerein-loaded liposomes and then spliced them onto ChS, thus creating a nanoparticle with anti-inflammation and targeting functions. Further, in 2022, Hsc, HC. et al. (Hsu et al., 2022) demonstrated that ChS could enhance chondrocytes proliferation and migration via inhibiting AKT/NF-κB pathway and inducing β-catenin. Taken together, ChS is a promising chondrocyte-targeted nanodrug. Wang, L. et al. (Wang et al., 2020) cultured Kashin-Beck disease (KBD) chondrocytes with nano-Se (SeCS) and consequently found that SeCS could reduce the apoptosis of KBD chondrocytes with the upregulation of carbohydrate sulfotransferase 12 and 15 (CHST-12, 15) uronyl 2-O-sulfotransferase (UST) on protein and mRNA level. The scholars firstly demonstrated that the concentration of Se in RA, OA, and KBD is significantly lower than that in control, indicating that the deficiency of Se may be a cause of RA, OA, and KBD. They treated KBD chondrocytes with SeCS and showed that the viability and ultrastructure were improved. The Western blot and q-PCR showed that CHST-12, 15 and UST were obviously upregulated on both protein and mRNA levels. It shows SeCS may recue the KBD/OA chondrocytes via modulating the expression of CHST-12, 15 and UST in them. D'Atri, D. et al. (D'Atri et al., 2021) proved that NGs (nanoghosts) could be both a multifunctional DDS and a targeting medication towards inflammatory chondrocytes in OA via a proof-of-concept experiment. A recent research studied by Baek, A. et al. (Baek et al., 2021) has shown that 3′-Sialyllactose (3′-SL) could reduce arthritis induced by IL-1β via various pathways, including reducing the level of ROS, inhibiting apoptosis of chondrocytes genetically, promoting chondrocytes synthesizing and secreting matrix components. It has been previously studied that a nuclear receptor, Retinoid X receptor α (RXRα), is widely expressed in chondrocytes (Collins-Racie et al., 2009; Ratneswaran et al., 2017). Thus, some scholars demonstrated that RXRα modulator K-80003 could alleviate the degradation of cartilage and synovium inflammation utilizing the property. (Li H et al., 2021).

Recently, the inherent properties of targeting, biocompatibility, and specific bio-functions of nucleic acid are drawing attention in the field. Current studies have proposed using circular RNAs (circRNAs) as targeted drugs that can inhibit the genes associated with the process of OA. Shen, S et al. (Shen et al., 2019) demonstrated that the circRNA (CircSERPINE2) could target the OA chondrocytes via targeting miR-1271-5p and E26 transformation-specific-related genes (ERG). The scholars firstly confirmed that CircSERPINE2 was relatively low in OA cartilage, then they treated the cells with CircSERPINE2. Eventually, they concluded that the drug-treated group downregulated MMPs expression, which was related to the degradation during the OA process. Feng, M. et al. (Feng et al., 2021) found that circ_0020093 could ease the degradation and apoptosis brought by IL-1β, a key inflammatory factor. The scholars found that circ_0020093 and SPRY1 expressions declined in IL-1β-induced OA chondrocytes. Further, they demonstrated that circ_0020093 could upregulate the SPRY1 expression via targeting miR-23b inhibition, which could alleviate the apoptosis induced by IL-1β. In a word, circ_0020093 can target the miR-23b/SPRY1 axis in chondrocytes, leading to the prevention of OA apoptosis. Shen, S. et al. (Shen et al., 2021) concluded that circPDE4B could lower the degradation and upregulate the repairment of articular cartilage via a series of experiments. Like the study mentioned above, circPDE4B was also downregulated in OA chondrocytes. The researchers further demonstrated that circPDE4B could promote RIC8 guanine-nucleotide exchange factor A (RIC8A) degradation, both of them participated in OA process. Eventually, the scholars confirmed that circPDE4B/RIC8A could regulate p-38/MAPK signaling pathway in chondrocytes. The overexpression of circPDE4B would inhibit the pathway, resulting in the decline of MMP and enhancement of cell viability. Long non-coding RNA (lncRNA) induced by icariin is also confirmed to promote proliferation and repress the loss of chondrocytes in an inflammatory microenvironment. Cytoskeleton regulator RNA (CYTOR) is a kind of lncRNA, Wang, G. et al. (Wang et al., 2021) demonstrated that the CYTOR knockdown could reverse the anti-OA drug’s protective effect in chondrocytes, indicating CYTOR to be a promising chondrocyte-targeted nanodrug for OA.

### ECM Targeted Nanodrugs

ECM is secreted by chondrocytes which are the only kind of cells showing presence in articular cartilage and is mainly composed of fibers (collagen), proteoglycans, and glycoproteins (Carballo et al., 2017). Those macromolecules could be the specific ligands for targeting nanodrugs. In this section, we will primarily describe the proteoglycan and collagen-targeted nanodrugs (Table 2).

**TABLE 2:** ECM targeted nanomaterials.

Name	Type	Mechanism	Citation
WYRGRL	Drug delivery system	Specifically interact with COL-II	Rothenfluh et al. (2008)
COL-II antibody			(Cho et al., 2014; Cho et al., 2015)
mAV		Target the negative charge of the ECM	He et al. (2020)
RH-SLNs			Ebada et al. (2022)
RAPA@Lipo@HMs			Lei et al. (2022)
PLL		Stimulated by OA environment	Lan et al. (2020)
PPNP			Li H et al. (2021)

#### Collagen Targeted Nanodrugs

Collagen is the major fiber in the ECM of cartilage. More precisely, collagen II (COL-II) is the predominant one. Thus, various targeted drugs could bind with COL-II specifically. Previous studies have confirmed that a six amino acid peptide (sequence WYRGRL) has the properties of binding COL-II and conjugating with therapeutic molecules, thus fabricating an ECM-targeted nanodrug. The scholars utilized phage display of peptide library to select appropriate ligands of the ECM. Eventually, they obtained the purpose via the selection of COL-II targeted WYRGRL with five rounds of bioplanning. In addition, they sequentially demonstrated that the peptide could significantly enhance the target effect for OA cartilage (Rothenfluh et al., 2008). Antibodies to COL-II can also enhance the reactivity of cartilage-targeted nanodrugs with ECM. Cho, H. and his colleagues have deeply investigated the area. The team conjugated COL-II antibody with liposomal nanomaterial to diagnose and rehabilitate OA in 2013. They utilized nanosomes to encapsulate the fluorescence and conjugate COL-II antibody to detect the OA at an early stage (Cho et al., 2014; Cho et al., 2015). The above researches performed by Cho, H. et al. indicate that the nanomaterials can promisingly deliver therapeutic molecules towards COL-II, thus targeting the OA lesion.

#### Polysaccharide and Its Derivatives Targeted Nanodrugs

Glycosaminoglycan (GAG) is widely distributed in ECM of cartilage, including hyaluronic acid (HA), chondroitin sulfate (ChS), and so on. Negatively charged glycosaminoglycans are enriched in the cartilage ECM and form cross-linked proteoglycans to form a negatively charged network structure (Shapiro et al., 2002; Palmer et al., 2006). Some previous studies have demonstrated that the negative charge of ECM is a promising targeting lesion for OA treatment. Researchers targeted the negatively charged proteoglycans in ECM by modulating different nanomaterials’ zeta potential (Brown et al., 2019).

He, T. et al. successfully confirmed that nano-Avidin (mAv) covalently conjugating drugs can lead to fast penetration and long maintain time in cartilage via reversibly integrating with the aggrecans with a negative charge (He et al., 2020). What’s more, Ebada, HM. et al. and Lei, Y. et al. composited two cationic liposomes named rhein hydrophobic ion pairing integrated solid lipid nanoparticles (RH-SLNs) and rapamycin-liposome–incorporating hyaluronic acid-based HMs (RAPA@Lipo@HMs) as DDSs to target negatively charged ECM of cartilage (Ebada et al., 2022; Lei et al., 2022).

#### Other Multiple Components Targeted Nanodrugs

The microenvironment of arthritis cartilage is different from the normal. For instance, some enzyme and inflammatory factors may be upregulated, and the pH may be lower. Therefore, the scholars can synthesize new nanoparticles which have a stronger response to the arthritis cartilage. Lan, Q. et al. produced a novel nano-DDS including two specific motifs that target the OA lesion, a conventional anti-inflammatory drug, and a biomaterial scaffold that releases the drug continuously. The scholars utilized poly (2-ethyl-2-oxazoline)-poly (ε-caprolactone) (PLL) to endow the nanomaterial with pH-response. Then they conjugated a specific peptide substrate of MMP-13 enzyme to obtain MMP-response. Taken together, the nanomaterial could specifically release its loaded therapeutic molecules in OA lesions (Lan et al., 2020). An ROS-responsive drug release system, also called boronate-stabilized polyphenol-poloxamer (PPNP) assembled dexamethasone (DEX) nanodrug, was exploited by Li, X. et al., in 2021. PPNP was reported to obtain limited ROS-response. It can deliver drug molecules to OA lesions, then the relatively high level of ROS could break the PPNP delivery system, leading to a drug release. The scholars demonstrated that the nanomaterial was highly sensitive in a 37°C, 50 × 10^−6^ M H_2_O_2_ environment (high ROS microenvironment *in vitro*), leading to 85% releasement of DEX (Li X et al., 2021).

## Synovium Targeted Nanodrugs

### Synovium Cells Targeted Nanodrugs

**FIGURE 3:**
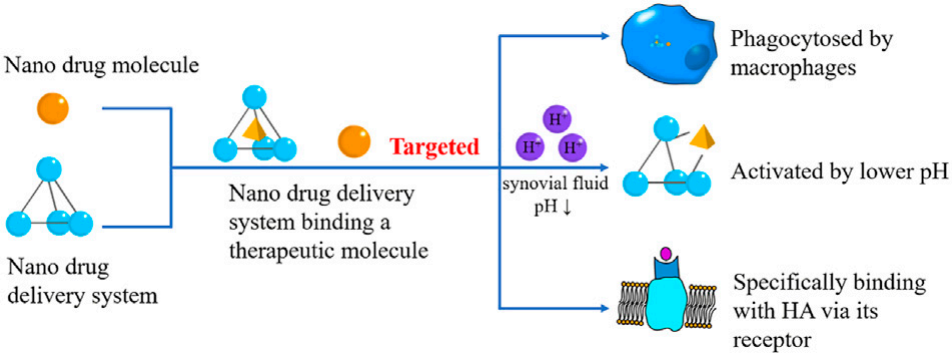
Synovium Targeted Nanodrugs. The synovium-targeted nanodrugs can also be classified into therapeutical molecules and DDSs. They can be phagocytosed by macrophages in the synovium, react with HA, or be activated by lower pH caused by OA.

Synovium consists of two layers which are divided into inner and outer ones. The inner one contains synovial cells, while the outer one gathers macrophages, fibroblasts, and capillaries (Mathiessen and Conaghan, 2017). Thus, nanomaterials can target the synovium in arthritis by exploiting the cells mentioned above (Figure 3) (Table 3).

**TABLE 3:** Synovium targeted nanomaterials.

Name	Type	Mechanism	Citation
Nano-liposomal preparation	Drug delivery system	Phagocytosed by macrophages and induce apoptosis via the NF-κB pathway	Barrera et al. (2000)
Nano-thiolated glycol chitosan packaged siRNA		Influence the polarization of macrophages via Notch 1 pathway	Kim et al. (2015)
miR365 antagomir/NPs-YCWP		Phagocytosed by macrophages, downregulated inflammatory factors and upregulated Nr1D2	Zhang L et al. (2020)
SOD-NPs		Endocytosed by synovial cells	Gui et al. (2022)
IL-Ra modified nanoparticle	Therapeutic molecule	Target synoviocytes via interacting with IL-1 receptors	Whitmire et al. (2012)
Vitronectin		Interact with FLSs via integrin-αβ	(Zhou et al., 2009; Ciregia et al., 2021)
Rh-PLGA-NPs@NH	Drug delivery system	Stimulated by OA environment	Hu et al. (2020)
PAA-MSNs			He M et al. (2021)

Plenty of DDSs have been explored to improve the drug treatment effect on synovium, such as polymers, polysaccharides, carbon-nanotubes, micelles, liposomes, and lipids (Brown et al., 2019). Macrophages play an important role in arthritis and are widely distributed in the synovium, mediating phagocytosis and polarization. Thus, macrophages are involved in the occurrence, development, and outcome of synovial inflammation in OA. In 2000, Barrera, P. et al. (Barrera et al., 2000) took advantage of the feature induced by macrophages, compositing nano-liposomal preparations that could be phagocytosed by macrophages and induce apoptosis via the NF-κB pathway. The researchers found that the injection of the clodronate liposomes results in the depletion of synovial macrophages. Then they discovered that the expression of intercellular adhesion molecule 1 (ICAM-1) and vascular cell adhesion molecule 1 (VCAM-1), two factors in synovitis, were downregulated after the clodronate liposomes treatment, further confirming the anti-inflammation effect via macrophage depletion. In 2015, Kim, MJ. et al. (Kim et al., 2015) used nano-thiolated glycol chitosan to package the siRNA, specifically modulating the Notch 1 pathway. The scholars demonstrated that the LPS-induced macrophages internalized the nanoparticles, the siRNA then specifically inhibited Notch 1 pathway, reducing the level of relevant mRNA (detected by real-time PCR). Thus, the activation of macrophages in the synovium is inhibited. In the same year, Jain, S. et al. (Jain et al., 2015) exploited alginate-decorated IL-10 plasmid nanoparticles which specifically bind macrophages in the synovium via Fc and neuropilin-1 receptors. The alginate-based nanoparticles could target macrophages, and then the plasmid could induce M2 polarization, alleviating arthritis. In 2020, Zhang, L. et al. (Zhang L et al., 2020) exploited a nano-tube delivery system mediated by yeast cell wall particles (YCWP) and loaded with miR365 antagomir, which could be specifically recognized and phagocytosed by macrophages after oral administration. *In vitro* experiment showed that macrophages successfully engulfed the miR365 antagomir/NPs-YCWP, sequentially downregulated inflammatory factors and upregulated Nr1D2. They also demonstrated that the nanoparticle could alleviate the OA of mice. Taken together, we can confirm that miR365 antagomir/NPs-YCWP could target synovial macrophages for OA therapy. In 2022, Gui, T. et al. exploited a superoxide dismutase-loaded porous polymersomes (SOD-NPs) that mainly accumulated in synovium tissue and reduced ROS production, further preventing the catabolism. The scholars firstly discovered that SOD-NPs were mainly distributed in synovial tissue *in vivo*. Then they demonstrated that synovial cells endocytosed SOD-NPs, leading to declining ROS, MMP, and other inflammatory factors. Thus, they confirmed that SOD-NPs could target OA synovial cells (Gui et al., 2022).

Nanoparticles can also directly react with targeting tissue, thus unleashing its treatment function. In 2012, Whitmire, RE. et al. (Whitmire et al., 2012) fabricated novel self-assembled nanoparticles, forming a submicron-scale structure that can bind interleukin-1 receptor antagonist (IL-Ra). The IL-Ra is the natural protein inhibitor of IL-1, thus could be a therapeutic molecule for OA. The scholars demonstrated that IL-Ra could specifically bind with IL-1 receptor on synoviocytes, endowing the nanoparticles with synoviocyte targeting and arthritis easement capacity. Inflamed synovium may show a higher presence of angiogenic endothelial cells, thus improving the level of integrin-α_v_β_3_. Early in 2009, Zhou, H. et al. (Zhou et al., 2009) demonstrated that nanoparticles modified with peptidomimetic vitronectin antagonists complementary with integrin-α_v_β_3_. While in 2021, Ciregia, F. et al. (Ciregia et al., 2021) confirmed that integrin-αβ was expressed on fibroblast-like synoviocytes (FLSs) as a receptor, allowing its ligand, vitronectin, to bind with itself specifically, thus mediating an arthritis-eliminating process.

### Synovial Fluid Targeted Nanodrugs

Synovial fluid is mainly secreted by synovial cells, consists of water, hyaluronic acid (HA), complement, polysaccharides, and cytokines. Multiple targeted nanomaterials have been endowed with the properties of high specificity towards synovial fluid and penetration through synovium layers (Table 3).

Synovial fluid contains a lot of HAs, an acidic mucopolysaccharide, lubricating joints and reducing the friction between articular cartilage. A review published by Altman, R et al. (Altman et al., 2019) has already shown that HA could specifically bind with its surface receptors, including CD44, toll-like receptor (TLR), ICAM-1, and layilin (LAYN). Murakami, T et al. (Murakami et al., 2019) previously corroborated that HA potentially reduces the apoptosis of chondrocytes via CD44, indicating CD44 could be an ideal targeted molecule for HA. Ragni, E. et al. (Ragni et al., 2019) demonstrated that CD44, a kind of receptor of HA, has the property of recruitment of extracellular vesicles (EVs) with CD44 markers. The statistics indicated that at the concentration of 2 mg/L of HA, the uptake of the EVs mediating by synoviocytes could be significantly improved. The research further explored the recruitment brought by the interaction between HA and CD44.

Like cartilage ECM, the composition of synovial fluid during OA changes, which leads to a lower pH and higher level of inflammatory factors (IL-1, IL-6, TNF, etc.). Hu, B. et al. (Hu et al., 2020) synthesized lactic-co-glycolic acid (PLGA) nanoparticles (NPs) loaded with rhein (Rh) and NHHCO (NH) (Rh-PLGA-NPs@NH). The Rh-PLGA-NPs@NH releases more therapeutic molecules in low pH synovial fluid environment which often shows in OA. He, M. et al. (He M et al., 2021) also designed a novel DDS consisting of pH-responsive polyacrylic acid (PAA) and mesoporous silica nanoparticles (MSNs). Therefore, the DDS could be more active in the acidic environment in OA, releasing more therapeutic molecules and improving the retention level of the medicine.

## Conclusion and Discussion

The exploitation of targeted nano-medicine for OA has been for several years. In these years, many researchers have synthesized different nanoparticles. These nanoparticles are used to construct DDSs for the targeted conveyance of medication or directly fabricate targeted nanodrugs. The above-mentioned two methods achieve the same goal through different approaches eventually increasing the concentration of the drug in the local lesion, the retention time of the drug, and reducing the side effects and the dosage of the drugs.

For targeted tissues, targeted nano-medicine for chondrocytes and cartilage matrix has been developed in large quantities. In contrast, the corresponding targeted nano-medicine for synovial matrix, synovial fluid, and synovial cells is still in urgent need of development. The main targets of ECM-targeted nanodrugs are collagen fibers and glycosaminoglycans, and collagen fibers are mainly COL-II. However, ECM contains a large amount of other collagen, so the development of these collagen fibers targeted nanodrugs still needs improvement.

When reviewing the literature in related fields, the author found the following problems. ①The synovial matrix and cartilage matrix are similar in composition. Can the targeted nano-drugs targeting the cartilage matrix also target the synovial matrix? ②The pH of synovial fluid and ECM can be lower in OA at the same time. Can the targeting of the drugs be carried out towards both sites?
